# Implementation of brief intervention for alcohol by Brazilian health professionals: overcoming difficulties

**DOI:** 10.1186/1940-0640-8-S1-A89

**Published:** 2013-09-04

**Authors:** Sonia Regina Zerbetto, Angélica Martins de Souza Gonçalves, Giselle Giovannetti, Denise Martins Gualtieri

**Affiliations:** 1 Department of Nursing, Federal University of São Carlos, São Carlos, São Paulo, Brazil; 2 Municipal Health of São Carlos, São Carlos, São Paulo, Brazil

## 

Training programs and training on prevention of alcohol issues for health professionals in primary health care must be constant, enabling the review of concepts, techniques and attitudes. This study aimed to identify the difficulties of healthcare professionals to implement Alcohol Screening and Brief Intervention in their health services, following completion of the training. This qualitative study was conducted with health professionals of units of the network of primary health care in a city in Sao Paulo, Brazil, undergoing training on standard screening instruments for alcohol consumption and implementation of BI through scheduled visits to their respective health units, and the data collected during the months of August to October 2012, through semistructured interviews. The data were subjected to content analysis of Bardin, thematic category, respecting the ethical standards of Resolution 196/96, as approved by the Research Ethics Committee, Opinion no. 53 021. The results showed two major themes with their respective subcategories: (1) The difficulties and problems encountered in Units, involving (1.1) human resources and infrastructure unit; (1.2) implementation of the screening Instrument for alcohol and brief intervention and (2) overcoming the difficulties and problems, seeking (2.1) the ways of improving dissemination of knowledge; (2.2) insertion strategies of the instrument and Brief Intervention in the health service. It was concluded that there is need for greater investment in continuing education to all health staff and in supervision, evaluation and ongoing monitoring of the process of implementing such strategies.

